# Comparison of Genetic Liability for Sleep Traits Among Individuals With Bipolar Disorder I or II and Control Participants

**DOI:** 10.1001/jamapsychiatry.2019.4079

**Published:** 2019-11-21

**Authors:** Katie J. S. Lewis, Alexander Richards, Robert Karlsson, Ganna Leonenko, Samuel E. Jones, Hannah J. Jones, Katherine Gordon-Smith, Liz Forty, Valentina Escott-Price, Michael J. Owen, Michael N. Weedon, Lisa Jones, Nick Craddock, Ian Jones, Mikael Landén, Michael C. O’Donovan, Arianna Di Florio

**Affiliations:** 1 Medical Research Council Centre for Neuropsychiatric Genetics and Genomics, Institute of Psychological Medicine and Clinical Neurosciences, School of Medicine, Cardiff University, Cardiff, United Kingdom; 2 Department of Medical Epidemiology and Biostatistics, Karolinska Institutet, Stockholm, Sweden; 3 Genetics of Complex Traits, University of Exeter Medical School, Exeter, United Kingdom; 4 Centre for Academic Mental Health, Population Health Sciences, Bristol Medical School, University of Bristol, Bristol, United Kingdom; 5 Medical Research Council Integrative Epidemiology Unit, University of Bristol, Bristol, United Kingdom; 6 National Institute of Health Research Biomedical Research Centre at University Hospitals Bristol NHS Foundation Trust and the University of Bristol, Bristol, United Kingdom; 7 Department of Psychological Medicine, University of Worcester, Worcester, United Kingdom; 8 National Centre for Mental Health, Cardiff University, Cardiff, United Kingdom; 9 Institute of Neuroscience and Physiology, Sahlgenska Academy at the Gothenburg University, Gothenburg, Sweden

## Abstract

**Importance:**

Insomnia, hypersomnia, and an evening chronotype are common in individuals with bipolar disorder (BD), but whether this reflects shared genetic liability is unclear. Stratifying by BD subtypes could elucidate this association and inform sleep and BD research.

**Objective:**

To assess whether polygenic risk scores (PRSs) for sleep traits are associated with BD subtypes I and II.

**Design, Setting, and Participants:**

This case-control study was conducted in the United Kingdom and Sweden with participants with BD and control participants. Multinomial regression was used to assess whether PRSs for insomnia, daytime sleepiness, sleep duration, and chronotype are associated with BD subtypes compared with control participants. Affected individuals were recruited from the Bipolar Disorder Research Network. Control participants were recruited from the 1958 British Birth Cohort and the UK Blood Service. Analyses were repeated in an independent Swedish sample from August 2018 to July 2019. All participants were of European ancestry.

**Exposures:**

Standardized PRSs derived using alleles from genome-wide association studies of insomnia, sleep duration, daytime sleepiness, and chronotype. These were adjusted for the first 10 population principal components, genotyping platforms, and sex.

**Main Outcomes and Measures:**

Association of PRSs with BD subtypes, determined by semistructured psychiatric interview and case notes.

**Results:**

The main analysis included 4672 participants with BD (3132 female participants [67.0%]; 3404 with BD-I [72.9%]) and 5714 control participants (2812 female participants [49.2%]). Insomnia PRS was associated with increased risk of BD-II (relative risk [RR], 1.14 [95% CI, 1.07-1.21]; *P* = 8.26 × 10^−5^) but not BD-I (RR, 0.98 [95% CI, 0.94-1.03]; *P* = .409) relative to control participants. Sleep-duration PRS was associated with BD-I (RR, 1.10 [95% CI, 1.06-1.15]; *P* = 1.13 × 10^−5^) but not BD-II (RR, 0.99 [95% CI, 0.93-1.06]; *P* = .818). Associations between (1) insomnia PRS and BD-II and (2) sleep-duration PRS and BD-I were replicated in the Swedish sample of 4366 individuals with BD (2697 female participants [61.8%]; 2627 with BD-I [60.2%]) and 6091 control participants (3767 female participants [61.8%]). Chronotype and daytime-sleepiness PRS were not associated with BD subtypes.

**Conclusions and Relevance:**

Per this analysis, BD subtypes differ in genetic liability to insomnia and hypersomnia, providing further evidence that the distinction between BD-I and BD-II has genetic validity. This distinction will be crucial in selecting participants for future research on the role of sleep disturbance in BD.

## Key Points

**Question:**

Does genetic liability to insomnia, hypersomnia, and chronotype differentiate subtypes of bipolar disorder?

**Findings:**

In this case-control study of 4672 participants with bipolar disorder and 5714 control participants, individuals with bipolar disorder I had significantly greater genetic liability to longer sleep duration, whereas individuals with bipolar disorder II had significantly greater genetic liability to insomnia; these findings were replicated in an independent sample. Individuals with bipolar subtypes did not differ in genetic liability to morning or evening chronotype.

**Meaning:**

Associations between polygenic liability to insomnia and hypersomnia and clinical strata within bipolar disorder are shown in this study for the first time, to our knowledge.

## Teaser

This case-control study uses a genome-wide association study and mendelian randomization to ascertain whether polygenic risk scores for sleep traits are associated with individuals with bipolar disorder I or II vs control participants.

## Introduction

Bipolar disorder (BD) and sleep have often been linked. First, reduced sleep duration, a symptom of manic episodes, has been implicated as a prodrome and trigger of mania.^1,2,3,4,5^ Second, insomnia (difficulty initiating and maintaining sleep^6^) and hypersomnia (prolonged sleep duration or excessive daytime sleepiness^7^) are commonly reported symptoms of bipolar depression^7,8,9^ that persist in the interepisode period^9,10,11,12,13,14,15,16,17,18^ and are associated with significant distress and impairment.^10^ Third, there is evidence that individuals with BD display greater evening preference for sleep (ie, an evening chronotype) than healthy control participants.^19,20,21^ At present, sleep interventions for individuals with BD primarily focus on reducing insomnia^22,23,24^ and stabilizing circadian rhythms.^25^ Understanding the association between sleep and BD is important and could inform clinical interventions.

Recent genome-wide association studies (GWAS)^26,27,28,29,30,31^ provide an opportunity to examine the association between sleep and BD at the genomic level. Using summary-level data, some studies have demonstrated a positive genetic correlation between BD and sleep duration.^28,30^ Other studies, however, have found no significant genetic correlations between BD, chronotype, and insomnia.^26,28^ These analyses have used summary-level data and therefore may have been limited by a lack of individual bipolar phenotypic and genotypic data. In particular, associations between BD subtypes^6^ (ie, type 1 [BD-I] and type 2 [BD-II]) and sleep traits have been neglected, despite evidence of heterogeneity between BD subtypes in genetics, illness course, clinical features, and etiologies.^32,33,34,35,36^ There is also evidence that individuals with BD subtypes differ in sensitivity to sleep loss^3^ and rates of hypersomnia and insomnia during depressive episodes.^37^

We therefore aimed to determine whether genetic liability for insomnia, hypersomnia, and chronotype differs in BD-I and BD-II. Given a lack of evidence on whether these sleep traits differ between individuals with BD-I or BD-II in the interepisode period, we had no prior hypothesis about which sleep traits, if any, would be associated with BD subtypes.

To test the associations between sleep and BD phenotypes, we adopted the polygenic risk score (PRS) method to estimate the burden of risk alleles associated with 4 sleep-associated phenotypes (insomnia, sleep duration, excessive daytime sleepiness, and chronotype) in people with BD-I or BD-II and control participants.^38,39^ In secondary analyses, we conducted a 2-sample mendelian randomization (MR) study to test whether the data were consistent with a causal association between sleep and BD phenotypes.

## Method

### Sample Recruitment

#### Individuals With BD

Participants with BD were recruited within the United Kingdom by the Bipolar Disorder Research Network (bdrn.org).^35^ All participants reported their race as white, were genetically unrelated, were 18 years or older, and had been recruited systematically (eg, via community mental health teams) or nonsystematically (eg, via websites, radio advertisements, or voluntary groups, such as Bipolar UK). Participants were excluded if they had affective illness experienced solely in response to alcohol or substance misuse or secondary to medical illness or medication use.

Participants provided written informed consent. The study had ethical approval from the West Midlands Multi-Centre Research Ethics Committee, in addition to local research and development approval by UK National Health Service Trusts and Health Boards.

#### Control Participants

Control participants aged 18 years or older were recruited via the UK Blood Service and the 1958 Birth Cohort. Characteristics and recruitment of this sample has been described previously.^40^ All control participants reported their race as white.

### Measures

Individuals with BD were assessed using the Schedules for Clinical Assessment in Neuropsychiatry interview,^41^ administered by trained research psychologists or psychiatrists in the research team (A.D.F., L.F., K.G.-S., L.J., N.C., and I.J.). Information from this interview was combined with clinical case note data to make lifetime best-estimate *DSM-IV* diagnoses. Measures taken to increase reliability of distinguishing BD subtypes are outlined in eAppendix 1 in the Supplement. Interrater reliability for differentiating between a best-estimate lifetime *DSM-IV* diagnosis of BD-I and BD-II was found to be good (κ, 0.85).

### Discovery Data Sets for Sleep Traits

The discovery data sets were GWAS summary statistics for insomnia,^27^ sleep duration,^30^ daytime sleepiness,^31^ and chronotype^42^ conducted in participants recruited to the UK Biobank.^43^ Sleep phenotypes were assessed using touchscreen questions. To assess insomnia symptoms, participants were asked, “Do you have trouble falling asleep at night, or do you wake up in the middle of the night?” with the possible responses “never/rarely,” “sometimes,” “usually,” and “prefer not to answer.” The insomnia GWAS was conducted in 236 163 participants who answered “usually” (affected individuals) or “never/rarely” (control participants). We chose to use this relatively extreme GWAS rather than a larger GWAS conducted by the same authors (ie, comparing responses of never or rarely with sometimes or usually for the question on insomnia symptoms) because (1) we considered this to better approximate meaningful insomnia; (2) it identified a larger number of genome-wide significant loci (28 vs 9 in the larger GWAS), suggesting that, despite the smaller sample size, a clearer distinction in phenotype offered better power; and (3) the authors used results of the extremes GWAS, not the larger GWAS, in validation analyses. The sleep-duration GWAS was conducted in 448 609 participants who were asked, “About how many hours sleep do you get in every 24 hours? (Please include naps.)” Responses were in 1-hour increments and were analyzed as a continuous variable. The daytime sleepiness GWAS was conducted in 452 071 participants and assessed using the question, “How likely are you to doze off or fall asleep during the daytime when you don’t mean to? (eg, when working, reading, or driving),” with responses “never/rarely,” “sometimes,” “often,” or “all the time” analyzed on a scale of 1 to 4 points.

The chronotype GWAS consisted of 403 195 individuals who answered the question “Do you consider yourself to be….?” Those who answered “definitely a ‘morning’ person” or “more a ‘morning’ than ‘evening’ person” were coded as affected individuals, and those who answered “more an ‘evening’ than a ‘morning’ person” or “definitely an ‘evening’ person” coded as control participants. Hence positive effect sizes from this GWAS indicate a morning chronotype, whereas negative effect sizes indicate an evening chronotype.

### Polygenic Risk Scores

Full details on genotyping, quality control, and imputation are in eAppendix 1 and the eFigure in the Supplement. We generated polygenic risk scores (PRSs) using PLINK version 1.9^44^ in PRSice.^45^ Imputed genotypes were clumped for linkage disequilibrium (window, 500 kb; *r*^2^ = 0.20), and single-nucleotide polymorphisms most significantly associated with sleep traits were retained. Clumping resulted in retaining 92 085, 92 096, and 91 950 single-nucleotide polymorphisms for daytime sleepiness, sleep duration, and insomnia, respectively. After clumping, PRSs for sleep traits were generated using PRSice^45^ at *P* value thresholds (P_T_) *P* < 1.00, *P* ≤ .50, *P* ≤ .20, *P* ≤ .10, *P* ≤ .05, *P* ≤ .01, and *P* ≤ .001 and converted to *z* scores. This range of *P* value thresholds was chosen in the absence of an independent sample that indicated which PRS *P* value threshold explained the most variance in each of the respective sleep phenotypes.

### Statistical Analysis

Data analysis was conducted in R version 3.33 (R Foundation for Statistical Computing). We performed multinomial logistic regression analyses examining associations between PRS for the aforementioned sleep traits (at the range of *P* value thresholds described) and individuals with BD subtypes (BD-I or BD-II) vs control participants. All analyses were adjusted for sex and 10 population principal components. In sensitivity analyses, we performed direct comparisons between the BD subtype groups by first performing the same multinomial regressions but changing the reference group from control participants to participants with BD-I or BD-II, and then by using logistic regressions that corrected for age, sex, and 10 population principal components. Results are reported at the *P* value thresholds that showed the most significant results with a false-discovery rate correction applied (using the Benjamini and Hochberg^46^ approach).

### MR Analyses

In cases in which we observed significant associations between sleep phenotype PRS and BD subtypes, we conducted follow-up 2-sample MR studies to test whether sleep phenotypes (exposures) were potentially causally related to BD subtypes (the outcome). Mendelian randomization is a causal inference method that uses genetic variants as instrumental variables for an exposure of interest. It relies on 3 assumptions: (1) genetic variants must be strongly associated with the exposure, (2) genetic variants should not be associated with confounders of the exposure-outcome relationship, and (3) genetic variants should only be associated with the outcome through the exposure in question.^47^ We used genome-wide significant single-nucleotide polymorphisms as genetic instruments for the sleep phenotypes. Instrument-exposure effects were taken from the sleep-trait GWAS summary statistics, and instrument-outcome effects were taken from BD-I and BD-II GWAS summary statistics.^48^ Four MR methods were used to assess relationships between sleep phenotypes and BD subtypes: the inverse variance weighted,^49^ weighted median,^50^ weighted mode,^51^ and MR Egger^49^ regression methods. To test for evidence of pleiotropy, we examined the intercept of MR Egger regressions^49^ and the Cochran *Q* and Rücker *Q* statistics.^52,53^ Data pruning, harmonization, and analyses were conducted in R version 3.33 using the “TwoSampleMR” package.^54^

### Replication Sample

We sought to replicate the study findings using Swedish individuals with BD (n = 4366) and control participants (n = 6091) recruited via the St Göran Bipolar project^55^ and the Swedish National Quality Register for Bipolar Affective Disorder (BipoläR).^56,57^ Full details of the samples, genotyping, quality control, and imputation are in eAppendix 2 in the Supplement.

## Results

### Sample Description

Among the individuals with BD, 3132 were female (67.0%), with a median age of 46 (range, 18-89) years. A total of 3404 participants met criteria for BD-I, and 1268 met criteria for BD-II. Among control participants, 2812 of 5714 (49.2%) were female. The Swedish sample consisted of 6091 control participants (3767 female participants [61.8%]) and 4366 affected individuals (2697 female participants [61.8%]), of whom 2627 met criteria for BD-I and 1739 met criteria for BD-II.

### Correlations Between PRSs for Sleep Traits

Across all PRS *P* value thresholds, insomnia PRSs were negatively associated with sleep-duration PRSs (*r* range, −0.17 to −0.30; *P* < 1 × 10^−4^) and positively associated with daytime-sleepiness PRSs (*r* range, 0.04-0.10; *P* = .007 to *P* < 1 × 10^−4^). Sleep-duration PRSs were negatively associated with daytime-sleepiness PRSs (*r* range, −0.03 to −0.00), but these associations were not significant across all thresholds (range, *P* = .028-.916). Morningness PRSs were not significantly associated with PRS for insomnia, sleep duration, or daytime sleepiness.

### PRSs for Sleep Traits: Case-Control Analyses

Logistic regression comparing individuals with BD with control participants revealed that, across all PRS *P* value thresholds, case status was significantly associated with PRSs for sleep duration (odds ratio [OR], 1.07 [95% CI, 1.03-1.12]; *P* = 5.52 × 10^−4^; *P* = 5.52 × 10^−4^ with adjustment for false-discovery rate [PRS *P*_T_ < 1.00]) and daytime sleepiness (OR, 1.10 [95% CI, 1.06-1.15]; *P* = 2.31 × 10^−6^; *P* = 1.05 × 10^−5^ with adjustment for false-discovery rate [PRS *P*_T_ ≤ .01]) and negatively associated with morning chronotype (OR, 0.91 [95% CI, 0.88-0.95]; *P* = 1.86 × 10^−5^; *P* = 6.26 × 10^−5^ with adjustment for false-discovery rate [PRS *P*_T_ ≤ .05]) but not significantly associated with insomnia (OR, 0.98 [95% CI, 0.94-1.02]; *P* = .39 [PRS *P*_T_ < 1.00]; eTables 2-5 in the Supplement).

### PRS for Sleep Traits by BD Subtypes

Results at the most significant PRS *P* value thresholds (with corrected *P* values) are summarized. Results at other PRS *P* value thresholds (*P*_T_) are provided in eAppendix 1 and eTables 18 and 19 in the Supplement.

#### Insomnia PRS

Multinomial regressions comparing individuals with BD subtypes to control participants revealed that insomnia PRS was significantly associated with a decreased risk of BD-I at a PRS *P*_T_ of *P* < 1.00 and *P* ≤ .50, but significant associations were not seen at other *P* value thresholds (eTable 6 in the Supplement). At all *P* value thresholds, insomnia PRS was significantly associated with BD-II (relative risk [RR], 1.14 [95% CI, 1.07-1.21]; *P* = 8.26 × 10^−5^, *P* = .001 with false-discovery rate adjustment [PRS *P*_T_ ≤ .001]). Results at all *P*_T_ are shown in the Figure, A. In direct tests, insomnia PRS was significantly associated with BD-II compared with BD-I (RR, 1.16 [95% CI, 1.08-1.24]; *P* = 1.39 × 10^−5^; *P* = 1.95 × 10^−4^ with false-discovery rate adjustment; OR, 1.14 [95% CI, 1.07-1.22]; *P* = 6.81 × 10^−5^ [PRS *P*_T_ ≤ .001]; eTables 10-11 in the Supplement).

**Figure.**
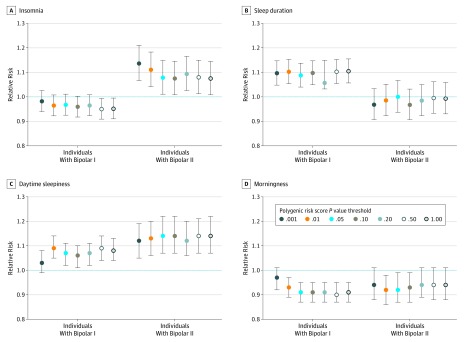
Relative Risk Ratios for Individuals With Bipolar Subtypes vs Control Participants Relative risk of insomnia (A), sleep duration (B), daytime sleepiness (C), and morningness (D) for patients with bipolar subtypes compared with control participants, as anticipated based on polygenic risk scores. Error bars indicate 95% CIs.

#### Sleep-Duration PRS

At all PRS *P* value thresholds, multinomial regression comparing individuals with BD subtypes with control participants revealed that sleep-duration PRS was associated with BD-I (RR, 1.10 [95% CI, 1.06-1.15]; *P* = 1.13 × 10^−5^; *P* = 1.07 × 10^−4^ with false-discovery rate adjustment [PRS *P*_T_ < 1.00]; eTable 7 in the Supplement). Associations between sleep-duration PRS and BD-II were not significant at any PRS *P* value threshold (eTable 7 in the Supplement). Results at all *P* value thresholds are shown in the Figure, B. Direct comparisons between the subgroups with BD-I and BD-II revealed that sleep-duration PRS was significantly associated with BD-I (RR, 1.11 [95% CI, 1.04-1.19]; *P* = 1.69 × 10^−3^; *P* = 4.74 × 10^−3^ with false-discovery rate adjustment; OR, 1.11 [95% CI, 1.04-1.19]; *P* = .002 [PRS *P*_T_ < 1.00]; eTables 12-13 in the Supplement).

#### Daytime-Sleepiness PRS

Compared with the control group, daytime-sleepiness PRS was associated with BD-I and BD-II at all PRS *P* value thresholds (except *P*_T_ < .001; eTable 8 in the Supplement). Results at all PRS *P*_T_ are shown in the Figure, C. Direct comparisons between BD subtypes were not significant after correction for multiple testing (eTable 14-15 in the Supplement).

#### Chronotype PRS

Polygenic risk score for morningness was associated with a reduced relative risk of BD-I in affected individuals compared with the control participants (RR, 0.90 [95% CI, 0.86-0.95]; *P* = 1.06 × 10^−5^; *P* = 1.11 × 10^−4^ with false-discovery rate adjustment [PRS *P*_T_ ≤ .50] at all PRS *P* value thresholds except *P*_T_ less than .001. In individuals with BD compared with control participants, morningness PRS was associated with a reduced risk of BD-II, but this finding was not significant across most PRS *P* value thresholds (eTable 9 in the Supplement). Results at all PRSs are shown in the Figure. Direct comparisons between BD subtypes were not significant (eTable 16-17 in the Supplement).

### MR Analyses

Across all MR methods, we did not find evidence of a potential causal relationship between insomnia and BD-II or sleep duration and BD-I. However, the direction of effect was consistent when assessing the effect of insomnia with BD-II (Table). Although MR Egger intercepts were not significantly different from zero for analyses of BD-I and BD-II , the Cochran *Q* and Rücker *Q* statistics indicated significant heterogeneity in effect estimates (insomnia: Rücker *Q*_35_ = 53.33; *P* = .024; Cochran *Q*_36_ = 54.03; *P* = .027; sleep duration: Rücker *Q*_54_ = 126.92; *P* = 8.33 × 10^−8^; Cochran *Q*_55_ = 128.91; *P* = 7.18 × 10^−8^; Table), possibly because of horizontal pleiotropy.

**Table.** Results of 2-Sample Mendelian Randomization Studies

Mendelian Randomization Method	Insomnia in Bipolar Disorder II^a^			Sleep Duration in Bipolar Disorder I^b^		
	log(Odds Ratio) or *Q* Statistic	SE or *df*	*P* Value	log(Odds Ratio) or *Q* Statistic	SE or *df*	*P* Value
Inverse variance weighted^c^	0.256	0.149	.087	0.396	0.209	.059
Weighted median^c^	0.355	0.183	.052	0.245	0.219	.264
Weighted mode^c^	0.410	0.300	.180	0.005	0.361	.990
MR Egger^c^	0.577	0.498	.255	-0.274	0.757	.719
Rücker *Q*^d^	53.33	35	.024	126.92	54	8.33 × 10^−8^
Cochran *Q*^d^	54.03	36	.027	128.91	55	7.18 × 10^−8^

^a^ 37 Single-nucleotide polymorphisms.

^b^ 56 Single-nucleotide polymorphisms.

^c^ Log(odds ratio) and SEs are presented.

^d^ *Q* statistics and *df* are presented.

### Replication Sample

In the Swedish sample, insomnia PRS was significantly associated with BD-II (RR, 1.07 [95% CI, 1.01-1.13]; *P* = .013 [PRS *P*_T_ ≤ .001]) compared with control participants, whereas the association with BD-I was not significant. Sleep-duration PRS was associated with a significant increased relative risk of BD-I compared with control participants (RR, 1.11 [95% CI, 1.06-1.16]; *P* = 1.72 × 10^−5^ [PRS *P*_T_ < 1.00]). The association between sleep-duration PRS and BD-II was marginally significant (RR, 1.06 [95% CI, 1.00-1.12]; *P* = .042 [PRS *P*_T_ <1.00]).

## Discussion

Bipolar disorder is heterogeneous in symptom presentation and most likely in the mechanisms that underlie these presentations. Genetics can help refine diagnostic groups that share similar etiologies.^58^ In this study, we provide what is to our knowledge the first evidence that genetic liability to insomnia and longer sleep duration differs according to BD subtype.

Genetic liability to insomnia as indexed by PRS was associated with increased relative risk of BD-II compared with control participants and those with BD-I. The stronger association between insomnia PRS and BD-II may explain nonsignificant genetic correlations between BD and insomnia in previous research,^26,28^ because individuals with BD-II are usually underrepresented in BD GWAS (eg, only 11% in a recent study^59^). Future research should explore possible reasons for this association.

Hypersomnia in BD populations has remained relatively underresearched, but researchers have recently called for increased efforts to understand its underlying biology and role in BD.^16,60^ This is because of its high prevalence and recurrence across bipolar depressive episodes^7,37^ in addition to high interepisode prevalence and association with relapse.^17,61^ We used sleep-duration and daytime-sleepiness PRS as proxies for genetic liability to hypersomnia. Sleep-duration PRS was associated with increased relative risk of BD-I but not BD-II and was significantly more strongly associated with BD-I than BD-II in a direct comparison. In contrast, daytime-sleepiness PRS was not significantly associated with BD subtypes. Daytime-sleepiness PRS may be a proxy for insomnia, in that daytime sleepiness can be induced by insomnia,^62^ and we observed significant positive correlations between insomnia and daytime-sleepiness PRS. These results support existing research on the importance of hypersomnia in individuals with BD^16,60^ (and BD-I in particular^37^) and provide further evidence that hypersomnia is not a unitary construct.^63,64^

The results of the MR analyses do not support potentially causal relationships between insomnia and BD-II or sleep duration and BD-I. However, we observed significant heterogeneity in the genetic instruments, thereby violating the third assumption of MR and potentially biasing the results. Therefore, while insomnia and sleep duration could be useful clinical stratifiers, there is currently insufficient evidence to support a causal inference. Further research is needed to elucidate the biological mechanisms underpinning the genetic association between BD-I and longer sleep duration.

### Implications

Clinical and biological heterogeneity, combined with a classification that is not grounded in biology, are obstacles to advancing BD research. We provide some evidence of heterogeneity in genetic propensity to some sleep traits within BD (specifically insomnia and sleep duration), highlighting differences in the way some sleep-associated genetic factors are associated with BD subtypes. This adds to previously published work on stratification in BD^65^ and work suggesting that different factors may influence the 2 conditions.^32,33,34^

These results suggest that clinical trials of sleep interventions should stratify participants by clinical subtype and genetic liability to insomnia or hypersomnia. Future work should explore which factors drive the differences in genetic liability for insomnia/sleep duration between BD subtypes.

### Strengths

This study was conducted on the world’s largest single cohort of BD with genotypic and phenotypic data. Phenotypic data were collected using face-to-face semistructured interviews and case notes with high interrater reliability. We were therefore able to explore genetic associations using individual-level genetic data, which provided more granularity than summary statistics. In addition, we were able to replicate the results for insomnia PRS and BD-II and sleep-duration PRS and BD-I in an independent sample.

### Limitations

This study has several limitations. First, potential recruitment bias in our BD sample may have reduced its representativeness and influenced the results.^66^ Second, we were unable to adjust for additional variables (eg, age, education), because these were unavailable for control participants. Third, the index of hypersomnia is imprecise because the available GWAS summary statistics measured sleep duration as total hours slept^30^; previous work suggests that hypersomnia is better characterized by total time in bed.^17,64,67^ Fourth, there is evidence that 5% to 17% of patients with BD-II convert to BD-I,^68,69^ which could have resulted in some individuals with BD-II being misclassified in this sample. However, this would have reduced power to observe differences between the 2 subtypes rather than resulted in positive results we observe for insomnia and sleep duration. Finally, variants associated with insomnia or hypersomnia at ages 40 to 69 years (the age of the UK Biobank sample^43^) may differ from those associated in childhood or early adulthood. This may have increased our type-2 error rate, because most patients with BD experience the first onset of impairing symptoms in adolescence or early adulthood.^70^ Genetic risk for insomnia or hypersomnia that manifests during or prior to early adulthood may be more strongly associated with BD than those associated with midlife insomnia. These results should be replicated using future sleep trait GWAS in younger samples of sufficient size for PRS analysis.

## Conclusions

To our knowledge, this is the first study to explore whether genetic liability for sleep traits is associated with clinical strata of individuals with BD. Future work should explore potential mechanisms underlying differences between the BD subtypes in genetic liability for sleep traits.

## Notes

Supplement. **eAppendix 1.** Bipolar Diagnoses, Genotyped Data, Additional Analyses and Replication Sample **eTable 1.** Description of genotyping platforms for the BD and control samples. SNP N is before QC and imputation. Wave 1 of the BDRN data is part of the International Cohort Collection for Bipolar Disorder (ICCBD). **eFigure.** Principal component analysis of imputed genotype data, after exclusion of variants showing platform frequency differences. **eTable 2.** Logistic regressions between polygenic risk scores for insomnia and bipolar disorder cases compared to controls **eTable 3.** Logistic regressions between polygenic risk scores for sleep duration and bipolar disorder cases compared to controls **eTable 4.** Logistic regressions between polygenic risk scores for daytime sleepiness and bipolar disorder cases compared to controls **eTable 5.** Logistic regressions between polygenic risk scores for morningness and bipolar disorder cases compared to controls **eTable 6.** Multinomial regressions between polygenic risk scores for insomnia and bipolar subtypes compared to controls. **eTable 7.** Multinomial regressions between polygenic risk scores for sleep duration and bipolar subtypes compared to controls. **eTable 8.** Multinomial regressions between polygenic risk scores for daytime sleepiness and bipolar subtypes compared to controls. **eTable 9.** Multinomial regressions between polygenic risk scores for morningness and bipolar subtypes compared to controls. **eTable 10.** Sensitivity analyses: Multinomial regressions of insomnia polygenic risk scores and clinical status using bipolar I disorder as the reference group. **eTable 11.** Logistic regressions between polygenic risk scores for insomnia and odds of bipolar II disorder compared to bipolar I disorder. **eTable 12.** Sensitivity analyses: Multinomial regressions of sleep duration polygenic risk scores and clinical status using bipolar II disorder as the reference group. **eTable 13.** Logistic regressions between polygenic risk scores for sleep duration and odds of bipolar I disorder compared to bipolar II disorder. **eTable 14.** Sensitivity analyses: Multinomial regressions of daytime sleepiness polygenic risk scores and clinical status using bipolar I disorder as the reference group. **eTable 15.** Logistic regressions between polygenic risk scores for daytime sleepiness and odds of bipolar II disorder compared to bipolar I disorder. **eTable 16.** Sensitivity analyses: Multinomial regressions of morningness polygenic risk scores and clinical status using bipolar II disorder as the reference group. **eTable 17.** Logistic regressions between polygenic risk scores for morningness and odds of bipolar I disorder compared to bipolar II disorder. **eTable 18.** Frequencies of cases and controls in Swedish replication sample by genotyping wave. **eAppendix 2.** Replication Sample - Materials and methods **eTable 19.** Frequencies of controls, bipolar I disorder cases, and bipolar II disorder cases (by sex). **eReferences.**
