# Supplementary material for: Comparison of Genetic Liability for Sleep Traits Among Individuals With Bipolar Disorder I or II and Control Participants
Source: JAMA Psychiatry. 2019 Nov 21;77(3):303–10. doi: 10.1001/jamapsychiatry.2019.4079 (PMC6902167; doi:10.1001/jamapsychiatry.2019.4079)
Supplement: Supplement. — eAppendix 1. Bipolar Diagnoses, Genotyped Data, Additional Analyses and Replication Sample eTable 1. Description of genotyping platforms for the BD and control samples. SNP N is before QC and imputation. Wave 1 of the BDRN data is part of the International Cohort Collection for Bipolar Disorder (ICCBD). eFigure. Principal component analysis of imputed genotype data, after exclusion of variants showing platform frequency differences. eTable 2. Logistic regressions between polygenic risk scores for insomnia and bipolar disorder cases compared to controls eTable 3. Logistic regressions between polygenic risk scores for sleep duration and bipolar disorder cases compared to controls eTable 4. Logistic regressions between polygenic risk scores for daytime sleepiness and bipolar disorder cases compared to controls eTable 5. Logistic regressions between polygenic risk scores for morningness and bipolar disorder cases compared to controls eTable 6. Multinomial regressions between polygenic risk scores for insomnia and bipolar subtypes compared to controls. eTable 7. Multinomial regressions between polygenic risk scores for sleep duration and bipolar subtypes compared to controls. eTable 8. Multinomial regressions between polygenic risk scores for daytime sleepiness and bipolar subtypes compared to controls. eTable 9. Multinomial regressions between polygenic risk scores for morningness and bipolar subtypes compared to controls. eTable 10. Sensitivity analyses: Multinomial regressions of insomnia polygenic risk scores and clinical status using bipolar I disorder as the reference group. eTable 11. Logistic regressions between polygenic risk scores for insomnia and odds of bipolar II disorder compared to bipolar I disorder. eTable 12. Sensitivity analyses: Multinomial regressions of sleep duration polygenic risk scores and clinical status using bipolar II disorder as the reference group. eTable 13. Logistic regressions between polygenic risk scores for sleep duration and odds o [file jamapsychiatry-77-303-s001.pdf]

Supplementary Online Content

Lewis KJS, Richards A, Karlsson R, et al. Comparison of genetic liability for sleep traits among individuals with bipolar disorder I or II and control participants. *JAMA Psychiatry*. Published online November 21, 2019. doi:10.1001/jamapsychiatry.2019.4079

eAppendix 1. Bipolar Diagnoses, Genotyped Data, Additional Analyses and Replication Sample

eTable 1. Description of genotyping platforms for the BD and control samples. SNP N is before QC and imputation. Wave 1 of the BDRN data is part of the International Cohort Collection for Bipolar Disorder (ICCBD).

eFigure. Principal component analysis of imputed genotype data, after exclusion of variants showing platform frequency differences.

eTable 2. Logistic regressions between polygenic risk scores for insomnia and bipolar disorder cases compared to controls

eTable 3. Logistic regressions between polygenic risk scores for sleep duration and bipolar disorder cases compared to controls

eTable 4. Logistic regressions between polygenic risk scores for daytime sleepiness and bipolar disorder cases compared to controls

eTable 5. Logistic regressions between polygenic risk scores for morningness and bipolar disorder cases compared to controls

eTable 6. Multinomial regressions between polygenic risk scores for insomnia and bipolar subtypes compared to controls.

eTable 7. Multinomial regressions between polygenic risk scores for sleep duration and bipolar subtypes compared to controls.

eTable 8. Multinomial regressions between polygenic risk scores for daytime sleepiness and bipolar subtypes compared to controls.

© 2019 Lewis KJS et al. *JAMA Psychiatry*.

eTable 9. Multinomial regressions between polygenic risk scores for morningness and bipolar subtypes compared to controls.

eTable 10. Sensitivity analyses: Multinomial regressions of insomnia polygenic risk scores and clinical status using bipolar I disorder as the reference group.

eTable 11. Logistic regressions between polygenic risk scores for insomnia and odds of bipolar II disorder compared to bipolar I disorder.

eTable 12. Sensitivity analyses: Multinomial regressions of sleep duration polygenic risk scores and clinical status using bipolar II disorder as the reference group.

eTable 13. Logistic regressions between polygenic risk scores for sleep duration and odds of bipolar I disorder compared to bipolar II disorder.

eTable 14. Sensitivity analyses: Multinomial regressions of daytime sleepiness polygenic risk scores and clinical status using bipolar I disorder as the reference group.

eTable 15. Logistic regressions between polygenic risk scores for daytime sleepiness and odds of bipolar II disorder compared to bipolar I disorder.

eTable 16. Sensitivity analyses: Multinomial regressions of morningness polygenic risk scores and clinical status using bipolar II disorder as the reference group.

eTable 17. Logistic regressions between polygenic risk scores for morningness and odds of bipolar I disorder compared to bipolar II disorder.

eTable 18. Frequencies of cases and controls in Swedish replication sample by genotyping wave.

eAppendix 2. Replication Sample - Materials and methods

eTable 19. Frequencies of controls, bipolar I disorder cases, and bipolar II disorder cases (by sex).

eReferences.

This supplementary material has been provided by the authors to give readers additional information about their work.

© 2019 Lewis KJS et al. *JAMA Psychiatry.*

eAppendix 1. Bipolar Diagnoses, Genotyped Data, Additional Analyses and Replication Sample

Bipolar Diagnoses

Section 10 of the Schedules for Clinical Assessment in Neuropsychiatry (SCAN) interview[41] was used to elicit symptoms of expansive mood. Other sections of the SCAN were used to elicit other aspects of psychopathology including depressive symptoms and psychotic symptoms. The information from interview and case notes was then combined to make clinical ratings. Psychiatric case notes were reviewed for 78% of participants. To reliably distinguish between BD-I and BD-II diagnoses: (1) diagnoses were informed by impairment criteria included in the diagnostic interview in addition to case note data, (2) all interviewers were research psychologists or psychiatrists based within the research team who undertook training to use the SCAN interview by senior members of the team who had previously undertaken training. Once trained, interviewers took part in ongoing audio reliability exercises., (3) senior members of the research team were involved in reliability meetings and consulted if there was any diagnostic uncertainty for particular cases.

© 2019 Lewis KJS et al. *JAMA Psychiatry.*

Genotyping, Quality Control, and Imputation

Genotyping was conducted on Affymetrix GeneChip 500K Mapping Array Set, Illumina Omni Express Array, and Illumina PsychChip. Strict quality control (QC) was performed separately on batches from each platform before merging. QC was conducted using PLINK version 1.9 software[1] in which single nucleotide polymorphisms (SNPs) were excluded if the minor allele frequency (MAF) was less than 0.01, if SNPs deviated from Hardy-Weinberg Equilibrium (HWE) at $P \leq 10^{-6}$ or call rate < 98%. Individuals were excluded from the sample if they had increased or decreased heterozygosity of |F| > 0.1, a discrepancy between their genotypic and reported sex, genotype call rate < 98%, high pairwise relatedness (pi-hat > 0.2) or did not cluster with European population samples in principal component analysis of 2000 participants from 19 populations of the 1000 Genomes Project.[2]

Exclusion of variants showing frequency differences between platforms principal component analysis (PCA) showed a strong effect of platform in the post-imputation dataset. To remove this effect, we performed pairwise association analyses between the three case batches, and another between the two control batches (eTable 1). Variants with association p<0.01 in any analysis were excluded (n=1,036,851). PCA performed after this step showed no effect of platform (eFigure 1).

© 2019 Lewis KJS et al. *JAMA Psychiatry.*

eTable 1. Description of genotyping platforms for the BD and control samples. SNP N is before QC and imputation. Wave 1 of the BDRN data is part of the International Cohort Collection for Bipolar Disorder (ICCBD).

	N cases	N controls	Chip	SNP N
**Wellcome Trust Case-Control Consortium (WTCCC)**	1868	2934	GeneChip 500K Mapping Array Set (Affymetrix)	377742
**Bipolar Disorder Research Network (BDRN) Wave 1**	2577	2784	Omni Express (Illumina)	393635
**Bipolar Disorder Research Network (BDRN) Wave 2**	1104	0	PsychChip (Illumina)	578318

© 2019 Lewis KJS et al. *JAMA Psychiatry.*

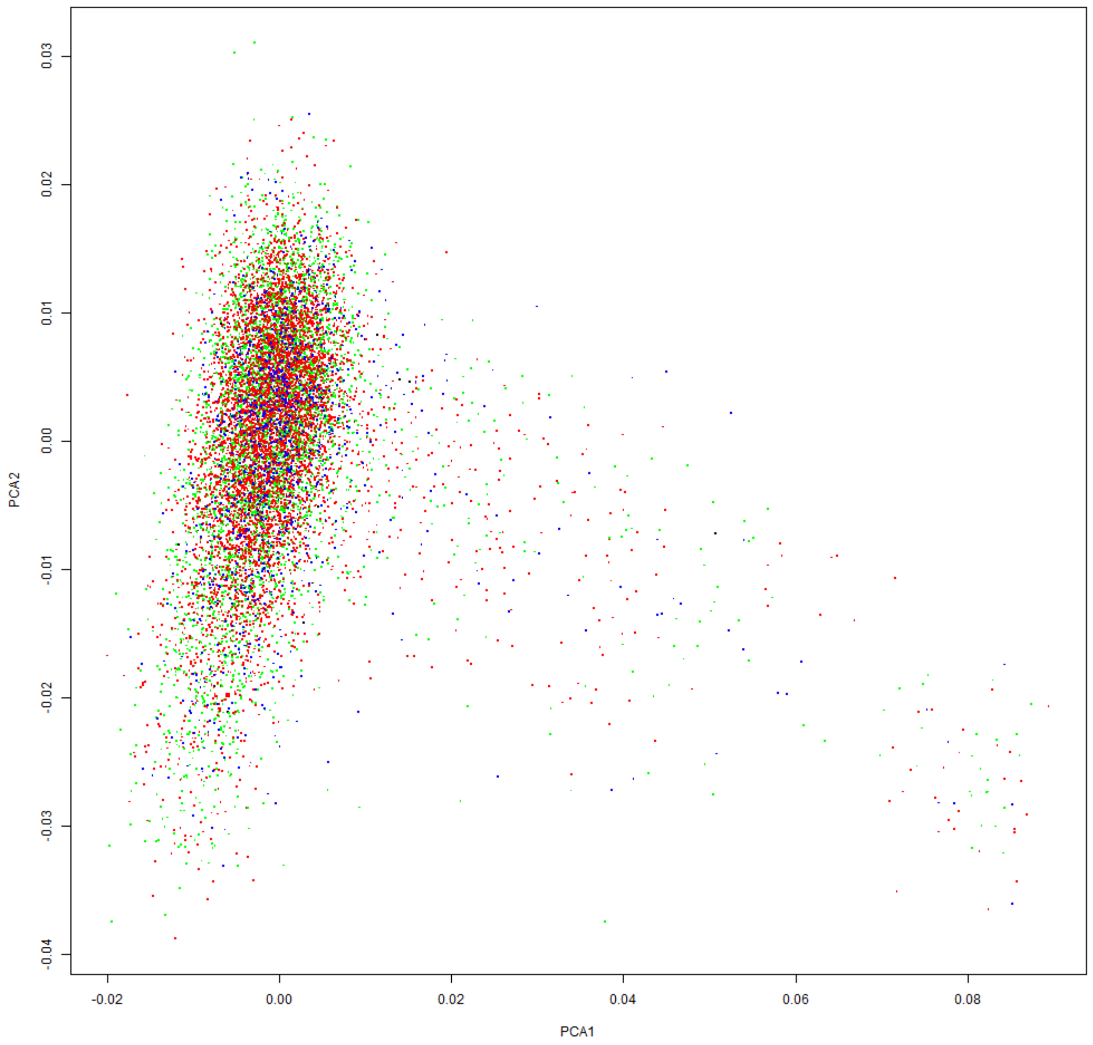

.

eFigure. Principal component analysis of imputed genotype data, after exclusion of variants showing platform frequency differences. Green — Wellcome Trust Case-Control Consortium samples, red— Bipolar Disorder Research Network wave 1, blue— Bipolar Disorder Research Network wave 2. See eTable 1 for descriptions of cases and controls in each sample.

After QC, data for each platform were phased using SHAPEIT version 3.4.0.1023[3] and imputed using IMPUTE2[4] with the 1000 Genomes Project reference panel (phase 3). Imputed data were converted to the most probable genotypes (probability ≥ 0.9) with additional SNPs excluded if the imputation INFO score was <0.8, MAF<0.01 or HWE *P* $<1x10^{-6}$). Imputed data were merged on shared SNPs. SNPs which showed large differences in frequency between samples on different genotyping platforms were removed.

© 2019 Lewis KJS et al. *JAMA Psychiatry.*

Control genotypic data was processed using the same method for QC, phasing and imputation as outlined for cases. After merging and removing SNPs with strand ambiguity, the final dataset used for analyses comprised 4,672 cases and 5,714 controls with 1,900,924 imputed SNPs.

Principal Component Analysis

PLINK version 1.9 software (Chang et al., 2015) was used to conduct principal component analysis on the clumped dataset. Eigenvectors for the first 10 principal components were included in all association analyses in order to control for potential confounding from population structure.

© 2019 Lewis KJS et al. *JAMA Psychiatry.*

eTable 2. Logistic regressions between polygenic risk scores for insomnia and bipolar disorder cases compared to controls

PRS pT	No. SNPs	OR	95% CI	*P*-value	*P*-value (FDR-adjusted)	Nagelkerke R^2
p ≤ 1	91950	0.98	0.94-1.02	0.389	0.917	8.83E-05
p ≤ .5	65942	0.98	0.94-1.02	0.393	0.917	8.68E-05
p ≤ .2	36927	1.00	0.96-1.04	0.874	0.986	2.98E-06
p ≤ .1	23718	0.99	0.95-1.03	0.562	0.983	4.00E-05
p ≤ .05	14652	0.99	0.96-1.04	0.799	0.986	7.73E-06
p ≤ .01	5415	1.00	0.96-1.04	0.986	0.986	3.58E-08
p ≤ .001	1410	1.02	0.98-1.06	0.358	0.917	1.00E-04

Analyses controlling for sex and 10 principal components. PRS pT = *P*-value threshold applied to discovery genome-wide association study in order to construct polygenic risk scores, OR = odds ratio, 95% CI = 95% Confidence Interval, FDR = False Discovery Rate.

© 2019 Lewis KJS et al. *JAMA Psychiatry.*

eTable 3. Logistic regressions between polygenic risk scores for sleep duration and bipolar disorder cases compared to controls

PRS pT	No. SNPs	OR	95% CI	*P*-value	*P*-value (FDR-adjusted)	Nagelkerke R^2
p ≤ 1	92096	1.07	1.03-1.12	5.52E-04	5.52E-04	1.42E-03
p ≤ .5	66188	1.07	1.03-1.12	6.38E-04	6.38E-04	1.39E-03
p ≤ .2	37493	1.07	1.03-1.11	0.001	0.001	1.20E-03
p ≤ .1	24321	1.06	1.02-1.11	0.004	0.004	1.01E-03
p ≤ .05	15240	1.06	1.02-1.11	0.003	0.003	1.05E-03
p ≤ .01	5867	1.07	1.03-1.12	0.001	0.001	1.25E-03
p ≤ .001	1676	1.06	1.02-1.11	0.004	0.004	9.65E-04

Analyses controlling for sex and 10 principal components. PRS pT = *P*-value threshold applied to discovery genome-wide association study in order to construct polygenic risk scores, OR = odds ratio, 95% CI = 95% Confidence Interval, FDR = False Discovery Rate.

© 2019 Lewis KJS et al. *JAMA Psychiatry.*

eTable 4. Logistic regressions between polygenic risk scores for daytime sleepiness and bipolar disorder cases compared to controls

PRS pT	No. SNPs	OR	95% CI	*P*-value	*P*-value (FDR-adjusted)	Nagelkerke R^2
p ≤ 1	92085	1.10	1.06-1.14	4.49E-06	1.05E-05	2.51E-03
p ≤ .5	65581	1.10	1.06-1.15	3.90E-06	1.05E-05	2.54E-03
p ≤ .2	35920	1.08	1.04-1.13	1.63E-04	2.28E-04	1.69E-03
p ≤ .1	22574	1.08	1.03-1.12	3.26E-04	3.80E-04	1.54E-03
p ≤ .05	13771	1.09	1.04-1.13	6.86E-05	1.20E-04	1.89E-03
p ≤ .01	4868	1.10	1.06-1.15	2.31E-06	1.05E-05	2.66E-03
p ≤ .001	1142	1.05	1.01-1.10	0.014	0.014	7.18E-04

Analyses controlling for sex and 10 principal components. PRS pT = *P*-value threshold applied to discovery genome-wide association study in order to construct polygenic risk scores, OR = odds ratio, 95% CI = 95% Confidence Interval, FDR = False Discovery Rate.

© 2019 Lewis KJS et al. *JAMA Psychiatry.*

eTable 5. Logistic regressions between polygenic risk scores for morningness and bipolar disorder cases compared to controls

PRS pT	No. SNPs	OR	95% CI	*P*-value	*P*-value (FDR-adjusted)	Nagelkerke R^2
p ≤ 1	91969	0.92	0.88-0.96	4.81E-05	6.73E-05	1.97E-03
p ≤ .5	67111	0.91	0.88-0.95	2.10E-05	6.26E-05	2.15E-03
p ≤ .2	39093	0.92	0.88-0.95	3.17E-05	6.26E-05	2.06E-03
p ≤ .1	26215	0.92	0.88-0.96	3.58E-05	6.26E-05	2.03E-03
p ≤ .05	17051	0.91	0.88-0.95	1.86E-05	6.26E-05	2.18E-03
p ≤ .01	7266	0.93	0.89-0.96	2.16E-04	2.51E-04	1.63E-03
p ≤ .001	2444	0.96	0.92-1.00	4.91E-02	4.91E-02	4.60E-04

Analyses controlling for sex and 10 principal components. PRS pT = *P*-value threshold applied to discovery genome-wide association study in order to construct polygenic risk scores, OR = odds ratio, 95% CI = 95% Confidence Interval, FDR = False Discovery Rate.

© 2019 Lewis KJS et al. *JAMA Psychiatry*.

eTable 6. Multinomial regressions between polygenic risk scores for insomnia and bipolar subtypes compared to controls.

		Bipolar I disorder				Bipolar II disorder			
PRS pT	No. SNPs	RR	95% CI	*P*-value	*P*-value (FDR-adjusted)	RR	95% CI	*P*-value	*P*-value (FDR-adjusted)
p ≤ 1	91950	0.95	0.91-0.99	**0.029**	**0.044**	1.07	1.01-1.14	**0.027**	**0.044**
p ≤ .5	65942	0.95	0.91-0.99	**0.025**	**0.044**	1.08	1.01-1.15	**0.019**	**0.044**
p ≤ .2	36927	0.96	0.92-1.01	0.112	0.130	1.09	1.03-1.17	**0.006**	**0.028**
p ≤ .1	23718	0.96	0.92-1.00	0.065	0.091	1.07	1.01-1.15	**0.027**	**0.044**
p ≤ .05	14652	0.97	0.93-1.01	0.142	0.153	1.08	1.01-1.15	**0.022**	**0.044**
p ≤ .01	5415	0.96	0.92-1.01	0.106	0.130	1.11	1.04-1.18	**0.001**	**0.009**
p ≤ .001	1410	0.98	0.94-1.03	0.409	0.409	1.14	1.07-1.21	**8.26E-05**	**0.001**

Analyses controlling for sex and 10 principal components. PRS pT = *P*-value threshold applied to discovery genome-wide association study in order to construct polygenic risk scores, RR = relative risk, 95% CI = 95% Confidence Interval, FDR = False Discovery Rate.

© 2019 Lewis KJS et al. *JAMA Psychiatry*.

eTable 7. Multinomial regressions between polygenic risk scores for sleep duration and bipolar subtypes compared to controls.

		Bipolar I disorder				Bipolar II disorder			
PRS pT	No. SNPs	RR	95% CI	*P*-value	*P*-value (FDR-adjusted)	RR	95% CI	*P*-value	*P*-value (FDR-adjusted)
p ≤ 1	92096	1.10	1.06-1.15	**1.13E-05**	**1.07E-04**	0.99	0.93-1.06	0.818	0.954
p ≤ .5	66188	1.10	1.05-1.15	**1.71E-05**	**1.07E-04**	1.00	0.93-1.06	0.886	0.954
p ≤ .2	37493	1.10	1.05-1.15	**3.05E-05**	**1.07E-04**	0.98	0.92-1.05	0.637	0.837
p ≤ .1	24321	1.10	1.05-1.15	**4.27E-05**	**1.20E-04**	0.97	0.91-1.03	0.316	0.510
p ≤ .05	15240	1.09	1.04-1.14	**2.26E-04**	**4.52E-04**	1.00	0.94-1.07	0.999	0.999
p ≤ .01	5867	1.10	1.05-1.15	**2.32E-05**	**1.07E-04**	0.99	0.92-1.05	0.658	0.837
p ≤ .001	1676	1.10	1.05-1.15	**6.46E-05**	**1.51E-04**	0.97	0.91-1.03	0.328	0.510

Analyses controlling for sex and 10 principal components. PRS pT = *P*-value threshold applied to discovery genome-wide association study in order to construct polygenic risk scores, RR = relative risk, 95% CI = 95% Confidence Interval, FDR = False Discovery Rate.

© 2019 Lewis KJS et al. *JAMA Psychiatry*.

eTable 8. Multinomial regressions between polygenic risk scores for daytime sleepiness and bipolar subtypes compared to controls.

		Bipolar I disorder				Bipolar II disorder			
PRS pT	No. SNPs	RR	95% CI	*P*-value	*P*-value (FDR-adjusted)	RR	95% CI	*P*-value	*P*-value (FDR-adjusted)
p ≤ 1	92085	1.08	1.04-1.13	**2.86E-04**	**4.55E-04**	1.14	1.07-1.22	**4.13E-05**	**1.65E-04**
p ≤ .5	65581	1.09	1.04-1.14	**2.14E-04**	**4.55E-04**	1.14	1.07-1.21	**5.79E-05**	**1.65E-04**
p ≤ .2	35920	1.07	1.02-1.11	**0.004**	**0.005**	1.12	1.06-1.20	**2.93E-04**	**4.55E-04**
p ≤ .1	22574	1.06	1.01-1.10	**0.016**	**0.017**	1.14	1.07-1.22	**4.59E-05**	**1.65E-04**
p ≤ .05	13771	1.07	1.02-1.11	**0.005**	**0.005**	1.14	1.07-1.22	**3.22E-05**	**1.65E-04**
p ≤ .01	4868	1.09	1.05-1.14	**5.89E-05**	**1.65E-04**	1.13	1.06-1.20	**2.70E-04**	**4.55E-04**
p ≤ .001	1142	1.03	0.99-1.08	0.199	0.199	1.12	1.05-1.19	**5.39E-04**	**7.55E-04**

Analyses controlling for sex and 10 principal components. PRS pT = *P*-value threshold applied to discovery genome-wide association study in order to construct polygenic risk scores, RR = relative risk, 95% CI = 95% Confidence Interval, FDR = False Discovery Rate.

© 2019 Lewis KJS et al. *JAMA Psychiatry.*

eTable 9. Multinomial regressions between polygenic risk scores for morningness and bipolar subtypes compared to controls.

		Bipolar I disorder				Bipolar II disorder			
PRS pT	No. SNPs	RR	95% CI	*P*-value	*P*-value (FDR-adjusted)	RR	95% CI	*P*-value	*P*-value (FDR-adjusted)
p ≤ 1	91969	0.91	0.87-0.95	**2.88E-05**	**1.16E-04**	0.94	0.88-1.01	0.078	0.086
p ≤ .5	67111	0.90	0.86-0.95	**1.06E-05**	**1.11E-04**	0.94	0.88-1.01	0.072	0.086
p ≤ .2	39093	0.91	0.87-0.95	**1.59E-05**	**1.11E-04**	0.94	0.88-1.01	0.080	0.086
p ≤ .1	26215	0.91	0.87-0.95	**4.55E-05**	**1.27E-04**	0.93	0.87-0.99	**0.031**	**0.048**
p ≤ .05	17051	0.91	0.87-0.95	**3.32E-05**	**1.16E-04**	0.92	0.87-0.99	**0.016**	**0.028**
p ≤ .01	7266	0.93	0.89-0.97	**0.001**	**0.002**	0.92	0.86-0.98	**0.009**	**0.018**
p ≤ .001	2444	0.97	0.92-1.01	**0.119**	**0.119**	0.94	0.88-1.00	0.070	0.086

Analyses controlling for sex and 10 principal components. PRS pT = *P*-value threshold applied to discovery genome-wide association study in order to construct polygenic risk scores, RR = relative risk, 95% CI = 95% Confidence Interval, FDR = False Discovery Rate.

© 2019 Lewis KJS et al. *JAMA Psychiatry*.

eTable 10. Sensitivity analyses: Multinomial regressions of insomnia polygenic risk scores and clinical status using bipolar I disorder as the reference group.

		Bipolar II disorder				Controls			
PRS pT	No. SNPs	RR	95% CI	*P*-value	*P*-value (FDR-adjusted)	RR	95% CI	*P*-value	*P*-value (FDR-adjusted)
p ≤ 1	91950	1.13	1.06-1.21	**3.13E-04**	**8.77E-04**	1.05	1.01-1.10	**0.029**	**0.044**
p ≤ .5	65942	1.14	1.06-1.21	**1.63E-04**	**7.23E-04**	1.05	1.01-1.10	**0.025**	**0.043**
p ≤ .2	36927	1.13	1.06-1.21	**2.07E-04**	**7.23E-04**	1.04	0.99-1.08	0.112	0.130
p ≤ .1	23718	1.12	1.05-1.20	**7.46E-04**	**1.74E-03**	1.04	1.00-1.09	0.065	0.091
p ≤ .05	14652	1.11	1.04-1.19	**1.38E-03**	**2.75E-03**	1.03	0.99-1.08	0.142	0.153
p ≤ .01	5415	1.15	1.08-1.23	**2.99E-05**	**2.09E-04**	1.04	0.99-1.08	0.106	0.130
p ≤ .001	1410	1.16	1.08-1.24	**1.39E-05**	**1.95E-04**	1.02	0.97-1.06	0.409	0.409

Analyses controlling for sex and 10 principal components. PRS pT = *P*-value threshold applied to discovery genome-wide association study in order to construct polygenic risk scores, RR = relative risk, 95% CI = 95% Confidence Interval, FDR = False Discovery Rate.

eTable 11. Logistic regressions between polygenic risk scores for insomnia and odds of bipolar II disorder compared to bipolar I disorder.

PRS pT	OR	95% CI	*P*-value	*P*-value (FDR-adjusted)	Nagelkerke R^2
p ≤ 1	1.12	1.05-1.20	**0.001**	**0.001**	3.19E-03
p ≤ .5	1.12	1.05-1.20	**5.65E-04**	**0.001**	3.51E-03
p ≤ .2	1.12	1.05-1.20	**6.19E-04**	**0.001**	3.46E-03
p ≤ .1	1.11	1.04-1.19	**0.002**	**0.002**	2.93E-03
p ≤ .05	1.11	1.04-1.18	**0.003**	**0.003**	2.61E-03
p ≤ .01	1.14	1.07-1.22	**6.98E-05**	**2.44E-04**	4.67E-03
p ≤ .001	1.14	1.07-1.22	**6.81E-05**	**2.44E-04**	4.68E-03

Analyses controlling for age, sex and 10 principal components. PRS pT = *P*-value threshold applied to discovery genome-wide association study in order to construct polygenic risk scores, OR = odds ratio, 95% CI = 95% Confidence Interval, FDR = False Discovery Rate.

© 2019 Lewis KJS et al. *JAMA Psychiatry.*

eTable 12. Sensitivity analyses: Multinomial regressions of sleep duration polygenic risk scores and clinical status using bipolar II disorder as the reference group.

		Bipolar I disorder				Controls			
PRS pT	No. SNPs	RR	95% CI	*P*-value	*P*-value (FDR-adjusted)	RR	95% CI	*P*-value	*P*-value (FDR-adjusted)
p ≤ 1	92096	1.11	1.04-1.19	**1.69E-03**	**4.74E-03**	1.01	0.94-1.07	0.818	0.954
p ≤ .5	66188	1.11	1.04-1.18	**2.73E-03**	**6.37E-03**	1.00	0.94-1.07	0.886	0.954
p ≤ .2	37493	1.12	1.04-1.19	**1.24E-03**	**4.35E-03**	1.02	0.95-1.08	0.636	0.837
p ≤ .1	24321	1.13	1.06-1.21	**2.29E-04**	**2.12E-03**	1.03	0.97-1.10	0.316	0.510
p ≤ .05	15240	1.09	1.02-1.16	**1.40E-02**	**2.79E-02**	1.00	0.94-1.07	0.999	0.999
p ≤ .01	5867	1.12	1.05-1.20	**1.20E-03**	**4.35E-03**	1.01	0.95-1.08	0.658	0.837
p ≤ .001	1676	1.13	1.06-1.21	**3.02E-04**	**2.12E-03**	1.03	0.97-1.10	0.328	0.510

Analyses controlling for sex and 10 principal components. PRS pT = *P*-value threshold applied to discovery genome-wide association study in order to construct polygenic risk scores, RR = relative risk, 95% CI = 95% Confidence Interval, FDR = False Discovery Rate.

© 2019 Lewis KJS et al. *JAMA Psychiatry*.

eTable 13. Logistic regressions between polygenic risk scores for sleep duration and odds of bipolar I disorder compared to bipolar II disorder.

PRS pT	OR	95% CI	*P*-value	P-value (FDR-adjusted)	Nagelkerke R^2
p ≤ 1	1.11	1.04-1.19	**0.002**	**0.003**	2.71E-03
p ≤ .5	1.11	1.03-1.18	**0.004**	**0.004**	2.50E-03
p ≤ .2	1.11	1.04-1.19	**0.002**	**0.003**	2.91E-03
p ≤ .1	1.13	1.06-1.21	**3.20E-04**	**0.001**	3.83E-03
p ≤ .05	1.09	1.02-1.16	**0.016**	**0.016**	1.71E-03
p ≤ .01	1.12	1.04-1.20	**0.001**	**0.003**	3.01E-03
p ≤ .001	1.13	1.06-1.21	**3.51E-04**	**0.001**	3.77E-03

Analyses controlling for age, sex and 10 principal components. PRS pT = *P*-value threshold applied to discovery genome-wide association study in order to construct polygenic risk scores, OR = odds ratio, 95% CI = 95% Confidence Interval, FDR = False Discovery Rate.

© 2019 Lewis KJS et al. *JAMA Psychiatry*.

eTable 14. Sensitivity analyses: Multinomial regressions of daytime sleepiness polygenic risk scores and clinical status using bipolar I disorder as the reference group.

		Bipolar II disorder				Controls			
PRS pT	No. SNPs	RR	95% CI	*P*-value	*P*-value (FDR-adjusted)	RR	95% CI	*P*-value	*P*-value (FDR-adjusted)
p ≤ 1	92085	1.05	0.99-1.12	1.24E-01	0.158	0.92	0.88-0.96	**2.86E-04**	**0.001**
p ≤ .5	65581	1.05	0.98-1.12	1.58E-01	0.185	0.92	0.88-0.96	**2.14E-04**	**0.001**
p ≤ .2	35920	1.05	0.99-1.13	0.111	0.155	0.94	0.90-0.98	**0.004**	**0.013**
p ≤ .1	22574	1.08	1.01-1.15	**0.020**	**0.035**	0.95	0.91-0.99	**0.016**	**0.032**
p ≤ .05	13771	1.07	1.01-1.15	**0.034**	0.052	0.94	0.90-0.98	**0.005**	**0.013**
p ≤ .01	4868	1.03	0.96-1.10	3.99E-01	0.399	0.91	0.87-0.96	**5.88E-05**	**0.001**
p ≤ .001	1142	1.09	1.02-1.16	0.013	0.031	0.97	0.93-1.02	0.199	0.215

Analyses controlling for sex and 10 principal components. PRS pT = *P*-value threshold applied to discovery genome-wide association study in order to construct polygenic risk scores, RR = relative risk, 95% CI = 95% Confidence Interval, FDR = False Discovery Rate.

© 2019 Lewis KJS et al. *JAMA Psychiatry.*

eTable 15. Logistic regressions between polygenic risk scores for daytime sleepiness and odds of bipolar II disorder compared to bipolar I disorder.

PRS pT	OR	95% CI	*P*-value	*P*-value (FDR-adjusted)	Nagelkerke R^2
p ≤ 1	1.05	0.99-1.12	0.130	0.182	6.75E-04
p ≤ .5	1.05	0.98-1.12	0.161	0.188	5.78E-04
p ≤ .2	1.05	0.99-1.13	0.114	0.182	7.38E-04
p ≤ .1	1.08	1.01-1.15	0.023	0.082	1.52E-03
p ≤ .05	1.07	1.00-1.15	0.038	0.090	1.26E-03
p ≤ .01	1.02	0.96-1.09	0.486	0.486	1.43E-04
p ≤ .001	1.08	1.01-1.15	0.020	0.082	1.59E-03

Analyses controlling for age, sex and 10 principal components. PRS pT = *P*-value threshold applied to discovery genome-wide association study in order to construct polygenic risk scores, OR = odds ratio, 95% CI = 95% Confidence Interval, FDR = False Discovery Rate.

© 2019 Lewis KJS et al. *JAMA Psychiatry.*

eTable 16. Sensitivity analyses: Multinomial regressions of morningness polygenic risk scores and clinical status using bipolar II disorder as the reference group.

		Bipolar I disorder				Controls			
PRS pT	No. SNPs	RR	95% CI	*P*-value	*P*-value (FDR-adjusted)	RR	95% CI	*P*-value	*P*-value (FDR-adjusted)
p ≤ 1	91969	0.96	0.90-1.03	0.278	0.389	1.06	0.99-1.13	0.078	0.160
p ≤ .5	67111	0.96	0.90-1.03	0.230	0.364	1.06	0.99-1.13	0.072	0.160
p ≤ .2	39093	0.96	0.90-1.03	0.234	0.364	1.06	0.99-1.13	0.080	0.160
p ≤ .1	26215	0.98	0.91-1.05	0.522	0.610	1.07	1.01-1.15	0.031	0.146
p ≤ .05	17051	0.98	0.92-1.05	0.645	0.694	1.08	1.01-1.15	0.016	0.114
p ≤ .01	7266	1.01	0.95-1.08	0.750	0.750	1.09	1.02-1.16	0.009	0.114
p ≤ .001	2444	1.02	0.96-1.09	0.478	0.608	1.06	1.00-1.13	0.070	0.160

Analyses controlling for sex and 10 principal components. PRS pT = *P*-value threshold applied to discovery genome-wide association study in order to construct polygenic risk scores, RR = relative risk, 95% CI = 95% Confidence Interval, FDR = False Discovery Rate.

© 2019 Lewis KJS et al. *JAMA Psychiatry*.

eTable 17. Logistic regressions between polygenic risk scores for morningness and odds of bipolar I disorder compared to bipolar II disorder.

PRS pT	OR	95% CI	*P*-value	*P*-value (FDR-adjusted)	Nagelkerke R^2
p ≤ 1	1.03	0.97-1.11	0.341	0.713	2.67E-04
p ≤ .5	1.04	0.97-1.11	0.298	0.713	3.19E-04
p ≤ .2	1.04	0.97-1.11	0.310	0.713	3.04E-04
p ≤ .1	1.02	0.95-1.09	0.614	0.716	7.49E-05
p ≤ .05	1.01	0.94-1.08	0.807	0.807	1.76E-05
p ≤ .01	0.98	0.92-1.05	0.576	0.716	9.22E-05
p ≤ .001	0.97	0.91-1.04	0.408	0.713	2.02E-04

Analyses controlling for age, sex and 10 principal components. PRS pT = *P*-value threshold applied to discovery genome-wide association study in order to construct polygenic risk scores, OR = odds ratio, 95% CI = 95% Confidence Interval, FDR = False Discovery Rate.

© 2019 Lewis KJS et al. *JAMA Psychiatry*.

eTable 18. Frequencies of cases and controls in Swedish replication sample by genotyping wave.

Genotyping wave	N cases	N controls	Chip	SNP N
sw34	1008	2158	Affymetrix 6.0	909622
swe6	1415	1271	Illumina OmniExpress	733202
lah1	3433	3070	Illumina PsychChip	574998

© 2019 Lewis KJS et al. *JAMA Psychiatry*.

eAppendix 2. Replication Sample - Materials and methods

Genotypes were generated in three waves. SNP and sample N are reported before QC and imputation.

QC was performed using the Ricopili pipeline (https://doi.org/10.1101/587196). Briefly, SNPs with missingness > 0.02, or case/control missingness difference > 0.02, or Hardy-Weinberg exact test p-value < 1e-6 (controls) or 1e-10 (cases), or minor allele frequency < 0.01 were excluded, as were samples with missingness > 0.02, or autosomal heterozygosity > 0.2 or < -0.2, or genotypic sex different from reported sex.

After QC, genotypes were aligned to the '+' strand, and imputed to the HRC 1.1 reference panel on the Sanger imputation server (http://www.ncbi.nlm.nih.gov/pubmed/27548312). Phasing was performed using EAGLE2, and imputation using PBWT.

PCA in imputed data was performed on combined best-guess genotypes using the Ricopili PCA module, excluding duplicated or closely related samples.

Before polygenic scoring, imputed data from the three genotyping waves were combined into one data set, and hard calls generated from genotype probabilities. In order to be in the combined genotype set, a SNP needed to be imputed with INFO >= 0.8, and to have 98% or more of individual genotype calls in each wave with a maximum posterior probability >= 0.9. As in the main analysis, indels, CNVs, multiallelic SNPs, and SNPS with strand-ambiguous allele coding were excluded.

SNPs with a p-value < 0.01 in any pairwise comparison between batches (stratified by case/control status and adjusted for 10 principal components) were removed before polygenic scoring (240478 SNPs).

In total, 3593273 SNPs were available for clumping and scoring.

Polygenic scoring was performed using PRSice version 2.2.2. Clumping and p-value thresholding parameters were the same as for the main analysis. Regression analyses were run in R version 3.6.0. Plink versions 1.90b4.9 and 2.00a2LM AVX2 Intel (28 Jun 2019) (https://doi.org/10.1186/s13742-015-0047-8), and GNU parallel (https://doi.org/10.5281/zenodo.1146014) were used for data preparation

© 2019 Lewis KJS et al. *JAMA Psychiatry.*

Cases with undetermined or missing subtype were excluded from both the subtype analyses and the case/control analyses. The frequencies of controls, bipolar I disorder cases, and bipolar II disorder cases (by sex) are displayed in eTable 16. Regression results were conducted for PRS vs bipolar subtype (multinomial logistic regression, controls as reference level). Multinomial regressions were adjusted for 10 principal components and sex.

© 2019 Lewis KJS et al. *JAMA Psychiatry.*

eTable 19. Frequencies of controls, bipolar I disorder cases, and bipolar II disorder cases (by sex).

	**Male**	**Female**
Control	2324	3767
BD-I	1125	1502
BD-II	544	1195

© 2019 Lewis KJS et al. *JAMA Psychiatry*.

eReferences

1. Chang CC, Chow CC, Tellier LCAM, Vattikuti S, Purcell SM, Lee JJ. Second-generation PLINK: rising to the challenge of larger and richer datasets. *Gigascience*. 2015;4(1):1-16.
2. The 1000 Genomes Project Consortium. A global reference for human genetic variation. *Nature*. 2015;526(7571):68-74. doi:10.1038/nature15393
3. Delaneau O, Marchini J, McVeanh GA, et al. Integrating sequence and array data to create an improved 1000 Genomes Project haplotype reference panel. *Nat Commun*. 2014;5:1-9. doi:10.1038/ncomms4934
4. Howie B, Fuchsberger C, Stephens M, Marchini J, Abecasis GR. Fast and accurate genotype imputation in genome-wide association studies through pre-phasing. *Nat Genet*. 2012;44(8):955-959. http://dx.doi.org/10.1038/ng.2354.

© 2019 Lewis KJS et al. *JAMA Psychiatry.*
